# Editorial of Special Issue “Tactile Sensing Technology and Systems”

**DOI:** 10.3390/mi11050506

**Published:** 2020-05-16

**Authors:** Maurizio Valle

**Affiliations:** Department of Electrical, Electronic, Telecommunications Engineering and Naval Architecture, University of Genova, Via Opera Pia 11A, I16145 Genova, Italy; maurizio.valle@unige.it

Human skin has remarkable features such as self-healing ability, flexibility, stretchability, high sensitivity and tactile sensing capability. It senses pressure, humidity, temperature and other multifaceted interactions with the surrounding environment. The imitation of human skin sensing properties via electronic systems is one of the frontrunner research topics in prosthetics, robotics, human-machine interfaces, artificial intelligence, virtual reality, haptics, biomedical instrumentation and healthcare, to name but a few.

Electronic skins or artificial skins are devices that aim to assimilate and/or mimic the versatility and richness of the human sense of touch via advanced materials and technologies. Generally, electronic skins encompass embedded electronic systems which integrate tactile sensing arrays, signal acquisition, data processing and decoding. 

Tactile sensors sense diversity of properties via direct physical contact (i.e., physical touch), e.g., vibration, humidity, softness, texture, shape, surface recognition, temperature, shear and normal forces. Tactile sensors are dispersed sensors that translate mechanical and physical variables and pain stimuli into electrical signals. They are based on a wide range of technologies and materials, e.g., capacitive, piezoresistive, optical, inductive, magnetic and strain gauges. Artificial tactile sensing allows the detection, measurement and conversion of tactile information acquired from physical interaction with objects. In the last two decades, the development of tactile sensors has shown impressive advances in sensor materials, design and fabrication, transduction techniques, capability and integration; however, tactile sensors are still limited by a set of constraints related to flexibility, conformability, stretchability, complexity and by real-time implementation of information decoding and processing. 

The Special Issue collects eight published papers, tackling the fabrication, integration and implementation of tactile sensing in some of the abovementioned applications such as haptics, robotics, human-computer interaction and artificial intelligence, modeling, decoding and processing of tactile information using machine learning techniques. 

Particularly, Wang et al. presented a flexible tactile sensor array (3x3) with surface texture recognition method for human-robotic interactions. They developed and tested a novel method based on Fine Element Modeling and phase delay to investigate the usability of the proposed flexible array for slippage and grooved surface discrimination when slipping over an object [1]. Choi et al. developed a skin-based biomimetic tactile sensor with bilayer structure and different elastic moduli to emulate human epidermal fingerprints ridges and epidermis. They proved that the proposed sensor has a texture detection capability for surfaces under 100µm with only 20µm height difference [2]. Chen et al. investigated the influence of skin thickness and thermal contact resistance on a thermal model for tactile perception. They proposed and tested a novel methodology to reproduce thermal cues for surface roughness recognition [3]. Kameoka et al. developed and assessed a pressure distribution sensor that measures stickiness when touching an adhesive surface via magnetic force offset [4]. Cordoba et al. proposed a set of calibration methods, Quasi Single Point Calibration Method (QSPCM), other than two-point calibration method (TPCM) for high-speed measurements of resistive sensors. The FPGA implementation of the proposed circuit has been used to quantify resistances values in the range (267.56 W,7464.5 W) [5]. Byun et al. presented a new gesture recognition method implemented on Flexible Epidermal Tactile Sensor FETSA based on strain gauge to sense object deformation. They prototyped and implemented a wearable hand gesture recognition smart watch. The latter demonstrated its ability to detect eight motions of the wrist and showed higher performance than preexisting arm bands, in terms of robustness, stability and repeatability [6]. Maurel et al. developed a viso-tactile substitution system based on vibrotactile feedback called TactiNET for the active exploration of the layout and the typography of web pages in a non-visual environment, the idea being to access the morpho-dispositional semantics of the message conveyed on the web. They evaluated the ability of the TactiNET to achieve the categorization of web pages in three domains—namely tourism, e-commerce and news [7]. Finally, Alemeh et al. assessed a comparison of embedded machine learning techniques—specifically convolutional neural networks models—for tactile data decoding units on different hardware platforms. The proposed model shows a classification accuracy around 90.88% and outperforms the current state-of art in terms of inference time [8]. 

I would like to take the opportunity to give my genuine thanks to all the authors for submitting such valuable scientific contributions to this Special Issue. Furthermore, my sincere undisputed thanks to all the reviewers for dedicating time and effort to revising the various manuscripts. 
